# NHLBI support of systems biology

**DOI:** 10.3389/fphys.2013.00299

**Published:** 2013-11-15

**Authors:** Pankaj Qasba, Jennie Larkin

**Affiliations:** ^1^Blood Diseases Branch, Division of Blood Diseases and Resources, HHS, National Institutes of Health, National Heart, Lung, and Blood InstituteBethesda, MD, USA; ^2^Advanced Technologies and Surgery Branch, Division of Cardiovascular Sciences, National Institutes of Health, National Heart, Lung, and Blood InstituteBethesda, MD, USA

**Keywords:** NHLBI, NIH, funding opportunity, systems biology research, portfolio analysis

## Abstract

The National Heart, Lung, and Blood Institute (NHLBI) has recognized the importance of the systems biology approach for understanding normal physiology and perturbations associated with heart, lung, blood, and sleep diseases and disorders. In 2006, NHLBI announced the Exploratory Program in Systems Biology program, followed in 2010 by the NHLBI Systems Biology Collaborations program. The goal of these programs is to support collaborative teams of investigators in using experimental and computational strategies to integrate the component parts of biological networks and pathways into computational models that are based firmly on and validated using experimental data. These validated models are then applied to gain insights into the mechanisms of altered system function in disease, to generate novel hypotheses regarding these mechanisms that can be tested experimentally, and to then use the results of experiments to refine the models. This perspective reviews the history of dedicated systems biology programs at NHLBI and reviews some promising directions for future research in this area.

Systems biology aims to help us understand and predict the behavior of complex systems through a combination of experimental and computational approaches with each approach informing the other. Although high-throughput omic technologies may help inform a systems approach, such approaches alone do not constitute a systems biology research program. Similarly, while systems biology research requires computational modeling, neither does simply doing modeling encompass a systems biology program. Indeed, systems biology research requires both computational and experimental (often high-throughput) approaches in research program that complement and inform each other. Experiments measure the system and its key properties and responses, while computational models integrate the information and generate new testable hypotheses. This combination of experimental and computational approaches have been helping to improve the understanding of complex systems in heart, lung, and blood research for decades, and is particularly useful in understanding and even predicting the emergent properties of these systems, whose responses and characteristics may be greater than the sum of their parts. A critical early exemplar of the usefulness and impact of such approaches is Denis Noble's computational modeling of the cardiac action potential (Noble, 1962), which demonstrated that cardiac action potential results from interactions between multiple ion channels. His model helped form the basis of an active and growing cardiac simulation research community that continues to improve upon the models to refine their predictive ability and usefulness (Trayanova et al., 2012). These and other exemplars demonstrate the promise that the systems biology approach may hold to better understand complex systems, their dysfunction in disease, and support development of new therapies.

## Synopsis of NHLBI activities to date

Over the past 8 years, the National Heart, Lung, and Blood Institute (NHLBI) has invested in targeted programs to foster such integrated experimental and computational approaches in promising areas throughout the heart, lung, blood, and sleep research portfolio. Guided by input from a working group in 2004, “A Systems Biology Approach to Regulatory Networks in Heart, Lung, Blood and Sleep Research” that was chaired by Drs. Leroy Hood and Joseph Nadeau, NHLBI targeted its investment in systems biology to support research collaborations between computational and experimental researchers focused on specific research challenges.

The Request for Application (RFA) “NHLBI Exploratory Program in Systems Biology (R33),” HL06-004 and HL07-005, was designed to foster these multi-disciplinary collaborations that required integration of different types of expertise (predictive computational models, informatics, and experimental systems). To emphasize the importance of the balance and integration of these different approaches to the overall project, this program supported independent, linked R33 awards to the different collaborating investigators in the project. This multiple linked award approach was necessary as this program pre-dated the National Institutes of Health Multiple Principle Investigator policy (http://grants.nih.gov/grants/guide/notice-files/NOT-OD-07-017.html).

These linked R33 awards independently supported both the computational and experimental investigators while requiring them to work closely together to produce a single coherent, integrated project. The R33 mechanism was used to emphasize the high risk, innovative science this program intended to support. These R33 awards were not renewable, and successful projects were expected to be competitive for subsequent investigator-initiated research proposals. To allow research communities in different stages of readiness to respond, the RFA was designed with multiple receipt dates and funded projects starting in 2006, 2007, and 2008. In the past, others have provided excellent reviews of the systems biology (Chuang et al., 2010; MacIlwain, 2011; Hood and Tian, 2012), here we provide an overview of the NHLBI support and development of the systems biology field as it applies to heart, lung, and blood research.

The “NHLBI Exploratory Program in Systems Biology” program was successful in developing this field across NHLBI research areas. Although NHLBI originally set aside $24.3 million to support nine awards, a total of twelve projects were funded for the RFA, given the number of high-quality applications. Some examples of the diversity of research supported through this program included “Blood Systems Biology,” “Systems Biology of Sudden Cardiac Death,” to the “Neurogenesis of Cough.” Modeling approaches in these projects ranged from physics-based models, to agent-based models, to analysis of signaling networks. As this program encouraged multi-scale modeling, many of the proposals included multiple modeling approaches to address challenges at the different scales within the system. Following this successful RFAs -program, NHLBI continues to support this area by transitioning this program from RFA with a set-aside budget to an ongoing Program Announcement (PA), “NHLBI Systems Biology Collaborations (R01)”- PAR09-214. This PA was released in 2009 and had two receipt dates per year but retained a special emphasis panel for review. Between these targeted funding opportunities (RFA-HL-06-004, RFA-HL-07-005, and PAR09-214), NHLBI has committed $45.6 M between 2006 and 2012 to support systems biology research. NHLBI continues to support this PA through the renewal PAR12-138 that is active till 2015.

The purpose of this program was to build a community of researchers interested in developing, applying, and sharing systems biology approaches to the wide variety of research challenges represented across NHLBI research. This has been successful through both the original RFA program and through the various other programs supported from NHLBI. The success of this has also been shown through the large and vibrant group of HLBS researchers funded through the Interagency Modeling and Analysis Group's Multi-Scale Modeling program (IMAG/MSM). Indeed, the NHLBI systems biology program and the MSM programs have held joint meetings for the last 3 years, because of the shared research interests between the programs. These interactive meetings allow cross-cutting discussions across diverse research areas that help keep researchers informed about innovative new computational approaches of potential interest to their own research area.

**Figure 1:**
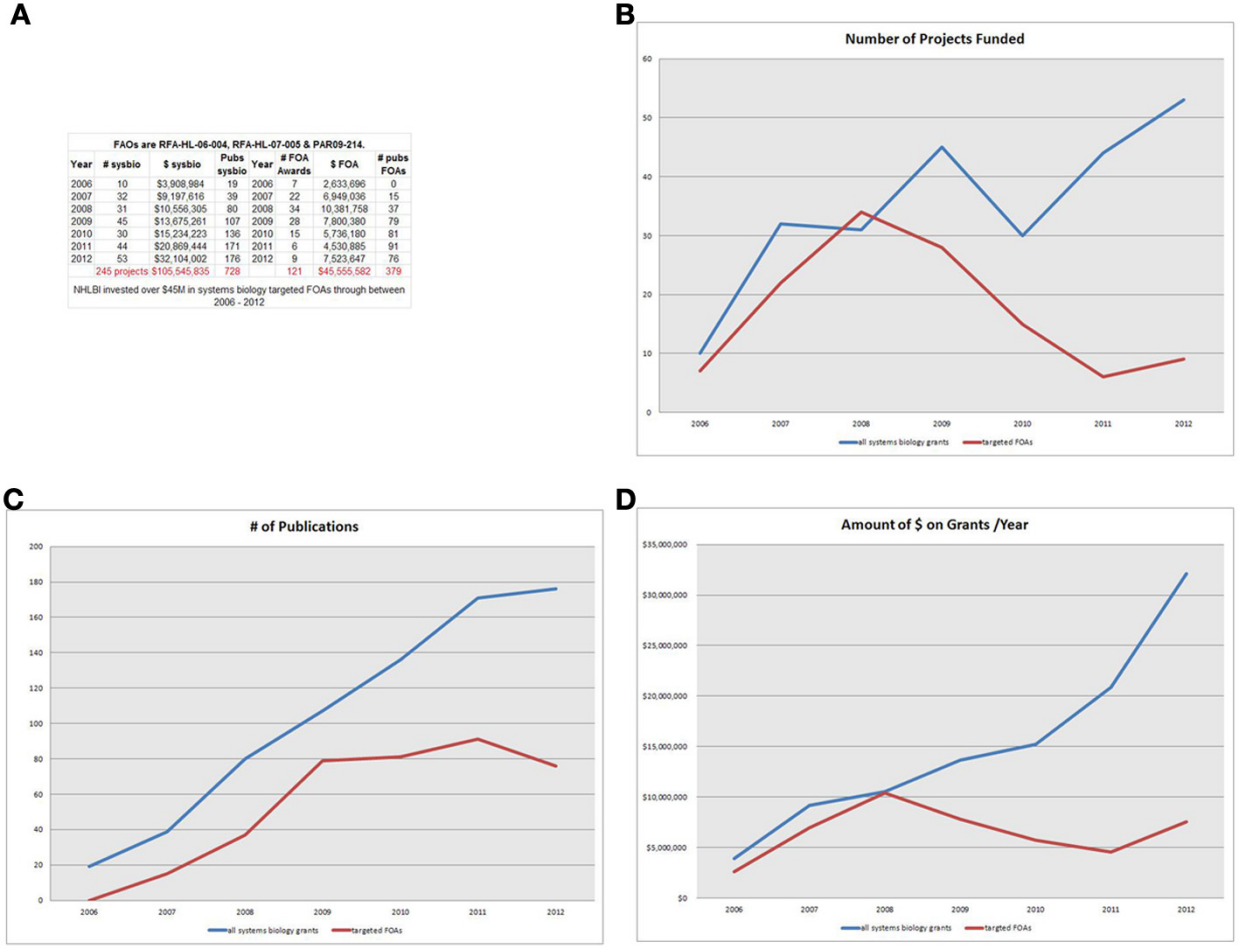
**(A)** NHLBI investment-Legend-NHLBI invested over $45 M in systems biology targeted FOAs through between 2006 and 2012. **(B)** # of projects-Legend-For the first three years, the targeted systems biology FOAs make up most of the awards in the NHLBI portfolio. But as RFA funding ends, the number of awards self-identifying as systems biology projects increases (2011 and beyond). **(C)** # of publications-Legend-Publications from the targeted FOAs gain impact in 1–3 year lag following funding, continuing to increase through 2011, although funding peaked for targeted FAOs in 2008. **(D)** Amount of $ on grants/year-Legend- Through 2008, virtually all funding goes to the RFA awards. However other (non-FOA) systems biology grants accelerate rapidly from 2009 to 2012.

An analysis of research funding of systems biology research using the NIH Reporter system (http://projectreporter.nih.gov/reporter.cfm) showed a marked growth in NHLBI systems biology research over the last 8 years (2006–2012). NHLBI support of research in this area through targeted announcements was assessed by searching the NIH Reporter database for grants funded through targeted funding opportunities RFA-HL-06-004, RFA-HL-07-005, or PAR-09-214. We determined number of awards, total costs, and number of publications in each year for each award. To determine the overall investment in the systems biology field, the same measures were taken for a search of grants that included the term “systems biology” in the title, abstract, or specific aims. This general search included only R21, R13, R33, and R01-equivalent grant mechanisms, excluding Program Projects (P01) and cooperative agreements. This analysis showed that the targeted funding announcements were responsible for the vast majority of systems biology projects supported by NHLBI from 2006 to 2009 (See Figure 1). However, starting in 2009, funding from these targeted announcements leveled off to about $5 M–$7 M per year, while the overall systems biology portfolio increased dramatically starting in 2009. Analysis of the output of the three targeted programs through publications shows an expected lag, with publications increasing from 2006 to 2009 then remaining steady, as it takes several years for the fruits of research to result in publications.

**Figure 2:**
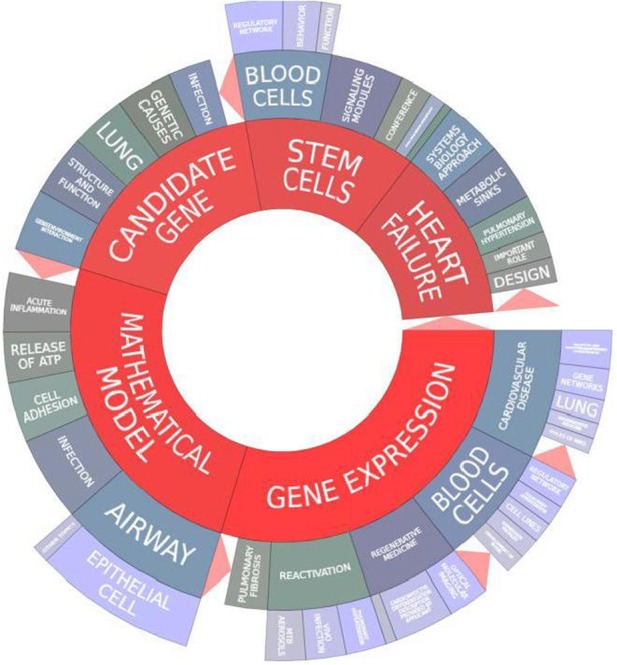
**NIH Reporter visualization of themes across the NHLBI systems biology portfolio (2006–2012)**.

A visualization of the key terms from the grants identified from the overall “systems biology” analysis from NIH Reporter identifies overall themes across the portfolio (See Figure 2). The most common terms were “mathematical model,” “gene expression,” “candidate gene,” “stem cells,” and “heart failure.” This analysis shows the diversity of scientific areas across the NHLBI portfolio using the systems biology approach: from heart failure, airway, and epithelial cell biology, to blood cell and stem cell regulation and infection. Approaches such as gene expression cut across disease areas, from pulmonary fibrosis, blood cells, to cardiovascular disease. This visualization gives a snapshot of the current NHLBI systems biology portfolio and highlights areas of strength and its diversity.

## Going forward

Going forward we hope to see continued growth of systems biology approaches to diverse research challenges, where they are useful and appropriate, as the integration of computational and biological/experimental methodologies can provide greater insight to complex biology. One area of particular interest is the potential of systems biology to help guide and accelerate translational research. For instance, an integrated systems level approach may prove useful in accelerating targeted drug development and reduced off-target drug effects (Sorger and Allerheiligen, 2011). Systems approaches may also help quantify behavioral influences on disease propensity. Such integration across physiological, genetic, and behavioral models is identified as a challenge in the current Interagency Multi-Scale Modeling Initiative, “Predictive Multiscale Models for Biomedical, Biological, Behavioral, Environmental and Clinical Research (Interagency U01)” (http://grants.nih.gov/grants/guide/pa-files/PAR-11-203.html).

Another developing area of interest is personalized precision medicine that promises individualized modeling to refine and target treatments to each person. While earlier examples of personalized medicine were seen in the field of pharmacogenomics (Jiayi et al., 2009), precision medicine includes a variety of different research areas, explicitly including mathematical modeling. For example in the cardiovascular arena, personalized models of the cardiovascular system are being developed that incorporate image-based information about the heart or major vessels from individual patients to generate predictive models tailored to that individual. Examples range from patient-specific models of cardiovascular mechanics (Krishnamurthy et al., 2013), to pre-surgical patient-specific aneurism models that help surgeons plan the surgery for each patient (Xiao et al., 2013), to application of individualized mathematical predictive models of blood flow in coronary arteries (Pijls and Sels, 2012), which has become a gold standard for invasive assessment of physiologic stenosis and an indispensable tool for decision making in coronary revascularization. Additionally, evidence that systems biology approaches have the potential to improve translation is the FDA approval of infusion pumps based on comprehensive models for diabetes. Models accurately recreated the precise and dynamic glucose-regulating function of a healthy pancreas (Kovatchev et al., 2009; Zhang et al., 2010).

Another area of interest is network-based approaches that seek to provide new insights into health and disease, by understanding complex interactions at the scale of individuals, tissues, or cells and the impact of disease state or therapeutics on disturbing these networks. One example is the Common Fund Library of Integrated Network based Cellular Signals (LINCS, http://www.lincsproject.org/) managed jointly by NHLBI and the National Human Genome Institute (NHGRI). LINCS aims to create a network-based understanding of biology by cataloging changes in gene expression and other cellular processes that occur when cells are exposed to a variety of perturbing agents and by using computational tools to integrate this diverse information into a comprehensive view of normal and disease states that can be applied for the development of new biomarkers and therapeutics. Another NHLBI program designed to utilize evolving knowledge of cellular and molecular networks to define common mechanism-associated traits across organ systems is Cross Organ Mechanism-Associated Phenotypes for Genetic Analyses of Heart, Lung, Blood, and Sleep Diseases (MAPGen, http://mapgenprogram.com/). The program identifies and characterizes common pathobiologic traits and/or mechanisms that cross organ systems and diseases with the ultimate goal of redefining heart, lung, blood, and sleep disorders based on newfound knowledge of the underlying molecular and/or cellular pathobiology. More recently, research in the MapGen program is making important strides to highlight our understanding of cellular networks and their relation to human diseasome, which may help foster network medicine (Barabasi et al., 2011; Chan and Loscalzo, 2012). Given the functional interdependencies between the molecular components in a human cell, a disease is rarely a consequence of an abnormality in a single gene but reflects the perturbations of the complex intracellular network (Loscalzo and Barabasi, 2011). The hope is that network medicine will provide a platform to methodically dissect molecular complexity of a particular disease. This may allow both identification of common molecular underpinnings of differing phenotypes, as well as identification of divergent molecular mechanisms that may underlie clinically similar phenotypes. Such molecular phenotypes will allow more precise diagnosis and more targeted therapies and treatments.

Similarly, another tool -genomic DNA footprinting- enables mapping of millions of *in vivo* binding sites for hundreds of transcription factors simultaneously in primary human cells (Lazarovici et al., 2013). The tools target a key bottleneck limiting construction of comprehensive regulatory networks for human cells typically requiring one-by-one or few-by-few discovery of connections between transcriptional regulators. The resulting maps enable the construction of accurate, comprehensive transcriptional regulatory networks that can, identify key regulatory factors for biological processes, help map networks associated with specific disease states and pinpoint specific regulatory factors that play a pathogenic role in disease (Maurano et al., 2012).

Finally, these emerging areas show the promise that systems biology approaches hold to help address key biomedical research challenges. The NHLBI programs have proven effective in fostering the development and application of these approaches across a wide range of areas, and we look forward to the day when researchers readily/keenly accept such quantitative approaches as part of a robust research strategy, just as DNA sequencing or RNA expression has become a common activity in biomedical research and not the sole domain of a few experts.

The views expressed by PQ and JL in this commentary are personal and do not necessarily represent those of the US Government.

### Conflict of interest statement

The authors declare that the research was conducted in the absence of any commercial or financial relationships that could be construed as a potential conflict of interest.
